# Seroprevalence of bovine coronavirus and factors associated with the serological status in dairy cattle in the western region of Thailand

**DOI:** 10.14202/vetworld.2021.2041-2047

**Published:** 2021-08-09

**Authors:** Samnang Ven, Pipat Arunvipas, Preeda Lertwatcharasarakul, Niorn Ratanapob

**Affiliations:** 1 Bio-Veterinary Science Program, Faculty of Veterinary Medicine, Kasetsart University, Kamphaeng Saen Campus, Kamphaeng Saen, Nakhon Pathom 73140, Thailand; 2 Department of Large Animal and Wildlife Clinical Sciences, Faculty of Veterinary Medicine, Kasetsart University, Kamphaeng Saen Campus, Kamphaeng Saen, Nakhon Pathom 73140, Thailand; 3 Department of Pathology, Faculty of Veterinary Medicine, Kasetsart University, Kamphaeng Saen Campus, Kamphaeng Saen, Nakhon Pathom 73140, Thailand

**Keywords:** bovine coronavirus, enzyme-linked immunosorbent assay, risk factor, seroprevalence

## Abstract

**Background and Aims:**

Bovine coronavirus (BCoV) is a pathogen affecting the productivities of dairy cattle worldwide. The present study aimed to determine the seroprevalence and factors associated with BCoV serological status using a commercial indirect enzyme-linked immunosorbent assay (ELISA).

**Materials and Methods:**

A cross-sectional study was conducted in the western region of Thailand. Blood samples were collected from 30 dairy herds. In total, 617 blood serum samples were tested using a commercial indirect ELISA for BCoV-specific immunoglobulin G antibodies. A questionnaire was used to collect data on the factors which have been identified as risk factors for BCoV antibody detection. The age and history of diarrhea of each animal were recorded. Fisher’s exact test was performed to univariately assess the association between BCoV serological status and possible risk factors. Variables with Fisher’s exact test p<0.10 were then evaluated using multivariate logistic regression to identify factors associated with BCoV serological status. The Bonferroni adjustment was used for multiple comparisons of significant variables in the final multivariate logistic regression model.

**Results:**

No herd was free from antibodies to BCoV. The individual seroprevalence of BCoV was 97.89% (604/617). The prevalence within herds was in the range of 45.45-100%. Cattle >3 years of age were more likely to be seropositive to BCoV compared to cattle <1 year of age (p=0.003), with the odds ratio being 81.96. Disinfecting diarrhea stools were a protective factor for being BCoV seropositive, with odds ratios of 0.08 and 0.06 compared to doing nothing (p=0.008) and to clean with water (p=0.002), respectively.

**Conclusion:**

BCoV seropositive dairy cattle were distributed throughout the western region of Thailand. The probability of being seropositive for BCoV increased with increasing animal age. Cleaning the contaminated stool with appropriate disinfectants should be recommended to farmers to minimize the spread of the virus.

## Introduction

Bovine coronavirus (BCoV) belongs to the beta-coronavirus genus clade A that is closely related to a coronavirus known as bovine-like coronavirus (BCoV-like) originating from captive wild ruminants [1]. Outbreaks of BCoV are considered relatively contagious and have occurred around the world [2,3]. BCoV is transmitted by the fecal-oral route, aerosols, and respiratory droplets [4], and infects the respiratory and gastrointestinal tracts of cattle of all ages. The infection leads to severe diarrhea, especially in dairy calves, with or without respiratory disease, especially during the winter season. In adult cattle, the infection can lead to severe to fatal outcomes when combined with other factors such as shipping stress or co-infections with other respiratory pathogens [5,6]. The disease may cause low mortality, but, the economic impacts are substantial, mainly due to significantly decreased milk production and body weight [7]. In addition, antibiotic use in infected animals causes financial losses from BCoV infection [8]. There are a few livestock and human coronavirus (CoVs) of significance, including the porcine epidemic diarrhea virus, transmissible gastroenteritis virus, and the novel CoV (SARS-CoV-2) which has been causing a remarkable global health challenge. Some of these CoVs have crossed species barriers. However, based on a recent experimental study, the susceptibility of cattle to SARS-CoV-2 infection was low. Moreover, there has been no evidence that SARS-CoV-2 can be transmitted from humans to cattle or vice versa [9]. In general, to detect enteric viruses, including BCoV, quantitative real-time polymerase chain reaction is commonly used [10]. The diagnosis of BCoV can be conducted using viral culture, antigen-captured enzyme-linked immunosorbent assay (ELISA), and hemagglutination assay [5]. According to reported sensitivity and specificity values, ELISA is the most suitable method to detect BCoV antibodies in cattle [2]. Important factors associated with the spread of BCoV within dairy herds are newly purchased animals and herd size. The prevalence is also varied by geographic location [6,11]. Animal age is associated with seroprevalence, which increases with increasing animal age [3]. Moreover, Workman *et al*. [12] found co-infection with other bacterial pathogens as one of the risk factors of BCoV infection.

According to a report of the Department of Livestock Development, almost half of the dairy cattle in Thailand are in the central and western parts of the country [13]. The main determinant of milk yield in Thai dairy herds is the genetics of the animals [14]. Infectious diseases, including BCoV, may also detrimentally affect milk production [4]. Thus, the disease status should be determined. A study in Thailand, focusing on developing a recombinant nucleocapsid protein ELISA (rN protein) for the detection of BCoV antibodies in dairy cattle, reported that 88% of cattle in the western and central regions of Thailand were positive [2]. However, factors associated with the serological status were not identified.

It would be of great interest to identify these factors to minimize losses caused by BCoV. Therefore, the present study aimed to determine the seroprevalence and factors associated with BCoV serological status using a commercial indirect ELISA.

## Materials and Methods

### Ethical approval

The study was approved by the Animal Care and Use for Scientific Research Committee, Kasetsart University, Bangkok, Thailand (ACKU62-VET-044). Farmers were willing to provide information about cattle and to permit blood sampling from their animals.

### Study period and location

**Figure-1:**
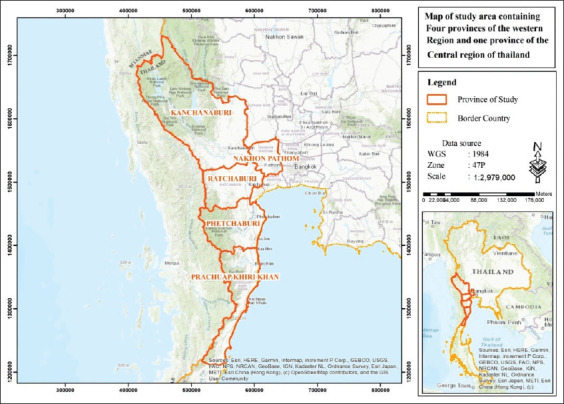
Study area containing five provinces in the western region of Thailand.

A cross-sectional study was conducted from May to September 2019, in dairy herds located in five provinces in the western region of Thailand (Figure-1). That was the rainy season in the country. The study was conducted during this period because of our convenience for sample collection. We expected that BCoV antibodies persist in animals for some time.

### Sample size determination

The sample size was calculated to determine the proportion of BCoV seropositive cattle, assuming an individual prevalence of 88%, based on a previous study in this area [2], with 95% confidence intervals, and 5% precision [15]. The sample size required was 163 heads. A number of samples was randomly selected from a total of 128,260 heads raised on 4,608 dairy herds located in the western region of Thailand [13]. Dairy cattle aged ≥6 months were conveniently selected from 30 dairy herds in five provinces.


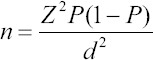


Where:

*n*=sample size

*Z*=Z statistic for a level of confidence,

*P*=expected prevalence or proportion, and

*d*=precision

### Sample collection

The 30 study herds were located in five provinces (Figure-1): Nakhon Pathom (n=11), Ratchaburi (n=10), Phetchaburi (n=3), Kanchanaburi (n=4), and Prachuap Khiri Khan (n=2). Blood samples were collected from the coccygeal vein and were transferred into 6.0 ml vacutainer tubes without anticoagulant. The age, physiological status, and history of diarrhea of each animal were recorded during sample collection. Samples were centrifuged at 15,000×*g* for 5 min. Serum samples were stored at −20°C until an indirect ELISA test was performed. A questionnaire was designed to be consistent with one of the objectives of the study, namely, to determine the possible factors associated with BCoV serological status such as herd size, housing, management, introducing new animals, and history of animal diarrhea in the herd.

### Commercial indirect ELISA antibody testing

All serum samples were evaluated using a commercial indirect ELISA antibody testing (SVANOVIR*^®^* BCV-Ab; Svanova, Sweden) for BCoV-specific immunoglobulin G (IgG) antibodies. The sensitivity and specificity of the kit are 84.6% and 100%, respectively. Serum samples were diluted at 1:25 in a dilution buffer to the plates coated with BCoV antigen. Negative and positive controls were included in every plate. Each sample was tested in duplicate. Secondary antibodies, goat anti-bovine IgG conjugated to horseradish peroxidase (HRP), were provided in the commercial kit. Reactions were developed using the TMB substrate system (3,3’,5,5’ tetramethylbenzidine) which links to the HRP enzyme at 18-25°C for 10 min. Then, a stop solution containing sulfuric acid was added to prevent fluctuation of the optical density (OD) value. The absorbance value of each well was read using an ELISA reader at a corrected OD >0.2 at 450 nm. The test was performed by following the manufacturer’s instructions. The results were interpreted based on the percentage positivity by dividing the sample OD values by the positive reference sample OD values. The cutoff value was set at 10%, according to the manufacturer’s instructions. Test validity was confirmed by the value of the corrected OD of the positive control being >0.5 and the percentage positivity of the negative control being <10%.

### Statistical analysis

All statistical analyses were carried out using the Stata 13 software (StataCorp LP, College Station, TX, US). Individual seroprevalence of BCoV was calculated. Variables obtained from the questionnaire and sampling records were examined using descriptive analysis. Variables having >15% missing values were not considered for further analyses. Fisher’s exact test was used as a univariate analysis to assess the association between BCoV serological status and each possible risk factor. Possible risk factors with p-values from univariate analysis <0.10 were then evaluated using multivariate logistic regression analysis. Variables with a p>0.05 were removed based on the backward stepwise method. The Bonferroni adjustment was used for multiple comparisons of significant variables in the final model.

## Results

In total, 617 blood samples were obtained from 30 dairy herds. The mean herd size of the participating herds was 56. All participating herds had not been vaccinated against BCoV. No animals included in the study presented any symptoms of BCoV infection during the sampling period. Every herd had at least one positive animal. Seroprevalence within herds was in the range of 45.45-100%. Approximately 98% of cattle (95% confidence interval; 96.7-99.2%) were positive for BCoV antibodies (Table-1).

**Table 1:** Seroprevalence of bovine coronavirus categorized by each variable and p-value obtained from Fisher’s exact test.

Variable	No. of negative	No. of positive	Prevalence (%)	p-value
Province*				
Nakhon pathom	9	140	93.95	0.009
Kanchanaburi	1	96	98.96	
Ratchaburi	1	188	99.47	
Phetchaburi	1	126	99.21	
Prachuap Khiri Khan	1	54	98.18	
History of diarrhea in herd				
Yes	13	498	97.45	0.139
No	0	106	100	
Barn type				
Tie stall	7	210	96.77	0.172
Tie stall+Free range	5	224	97.76	
Free range	1	175	99.43	
Herd size*				
Small (<20 heads)	3	32	91.42	0.004
Medium (>20-50 heads)	8	249	96.88	
Large (>50 heads)	2	322	99.38	
Introducing new animals				
Yes	5	202	97.58	0.769
No	8	402	98.04	
Age of farm*				
1-3 years	3	7	70.00	0.002
>3-5 years	1	49	98.00	
>5-10 years	0	19	100	
>10 years	9	529	98.32	
Rodents in farm				
Yes	13	497	97.45	0.139
No	0	107	100	
Pen division				
Yes	7	368	98.13	0.775
No	6	236	97.52	
Pets in farm*				
Yes	9	500	98.23	0.055
No	4	64	94.11	
Diarrhea stool management*				
Doing nothing	2	189	98.95	<0.001
Cleaning with water	2	350	99.43	
Using disinfectants	9	65	87.83	
Availability of feed storage space*				
Yes	12	413	97.17	0.074
No	1	191	99.47	
Source of water				
Running water	1	27	96.43	0.457
Natural water	12	577	97.96	
Colostrum intake				
Adequate	11	433	97.52	0.701
Inadequate	1	87	98.86	
Age of cattle*				
≤1 year	2	3	60.00	<0.001
>1-3 years	9	193	95.54	
>3 years	2	344	99.42	

* Variable having p-value<0.1 in univariate analysis

Seven factors met the criteria based on univariate analysis using Fisher’s exact test (Table-1). However, there was some collinearity among these variables. Thus, only three variables were included in the initial multivariate logistic regression model: Age of animal, diarrhea stool management, and age of farm. The final model with two significant variables is displayed in Table-2. The probability of being seropositive for BCoV increased with age, whereas disinfection of the diarrhea stools was a protective factor for BCoV antibody detection. According to the Bonferroni adjustment, the difference between cattle >3 years and ≤1 year was significant (p=0.003), as shown in Table-3, while the difference between >3 years and >1-3 years tended to be insignificant (p=0.090). Cattle >3 years had 81.96 times the odds of being positive to BCoV antibodies than cattle ≤1 year. Regarding diarrhea stool management, using disinfection was significantly better when compared to both doing nothing (p=0.008) and cleaning with water (p=0.002) with odds ratios of 12.29 and 15.94, respectively.

**Table 2:** Multivariate logistic regression model identifying factors associated with bovine coronavirus serological status.

Variable	Coefficient	p-value	OR	95%CI
Age of cattle				
<1 year				
>1-3 years	2.489	0.034	12.05	0.187-4.971
>3-5 years	4.406	0.001	81.96	1.804-7.008
Diarrhea stool management				
Doing nothing				
Cleaning with water	0.261	0.799	1.298	−1.746-2.268
Using disinfectants	−2.509	0.003	0.081	(−4.135)(−0.882)

OR=Odds ratio, CI=Confidence interval

**Table 3:** Multiple comparisons with the Bonferroni adjustment of variables associated with bovine coronavirus serological status in the final multivariate regression model.

Comparison	Coefficient	SE	p-value
Age			
>1-3 years versus ≤1 year	2.489	1.174	0.102
>3 years versus ≤1 year	4.406	1.328	0.003
>3 years versus >1-3 years	1.917	0.800	0.090
Diarrhea stool management			
Cleaning with water versus doing nothing	0.261	1.024	1.000
Using disinfectants versus doing nothing	−2.509	0.830	0.008
Using disinfectants versus cleaning with water	−2.769	0.813	0.002

SE=Standard error

## Discussion

A study in Saraburi Province, Thailand, reported 93% of dairy herds being positive to BCoV antibodies based on bulk tank milk samples [16]. This was lower than the herd seroprevalence found in the present study. The individual seroprevalence found in the present study was higher than in a previous study conducted in the western and central regions of Thailand that reported 88% (204/231) of cattle were positive to BCoV based on a commercial antibody ELISA test [2]. Because all herds were positive to BCoV antibodies, factors associated with the antibody detection at the herd level could not be determined. Based on the 100% herd seroprevalence and 98% individual seroprevalence found in the present study, BCoV should be considered as an endemic disease in this area. Therefore, the development of a local vaccine might be considered. The seroprevalence found in the present study was also high compared to studies in other countries [17-21]. In Norway, the lower prevalence was more likely due to younger animals being sampled [6]. In addition, the prevalence of BCoV can be affected by differences among studies in terms of animal husbandry, season, and geographical location [22,23].

A strong association between seroprevalence and age was identified by a previous study, which reported high seroprevalence of BCoV in older animals (>3-5 years of age) [19]. The positive association between BCoV serological status and age was probably due to the persistence of the infection [24]. Furthermore, Singasa *et al*. [2] suggested that the virus antibodies might still be detectable for years.

BCoV is sensitive to soap and disinfectants, though it can remain infectious for up to 3 days in soil, feces, and bedding materials [25]. In the present study, the use of disinfectants on diarrhea stools was associated with a lower probability of being seropositive for BCoV. Using water to clean the diarrhea stools was not helpful because it did not destroy the virus and might contribute to spreading the virus. Iodine and glutaraldehyde can be applied for pen hygiene, as they are effective in eliminating several microorganisms, especially pathogens which cause diarrhea, such as rotavirus, coronavirus, *Escherichia coli, Salmonella* spp., and *Cryptosporidium parvum* [26]. Contaminated vehicles, storage containers, and feeding equipment should be regularly cleaned using disinfectants to minimize the outspread of infectious diseases [27]. To avoid losses from enteropathogens including BCoV, proper hygienic practices should be routinely implemented in dairy herds [28]. Human hygiene in dairy herds can be addressed by the recommended use of decontaminating soap and alcohol.

Gomez *et al*. [29] reported variation in the prevalence of BCoV by year. A significant univariate association between serological status and age of the herd was found in the present study (Table-1). However, this variable was not significant in the multivariate analysis. Spending a longer period in the farming business was related to a higher risk of being seropositive for BCoV. This might have resulted from persistent infection caused by the virus [30]. In addition, a high concentration of viruses in feces along with their resistance in the environment may lead to permanent contamination of housing premises and consequently infection of animals in the herd [31].

There were four variables that were significantly associated with BCoV status based on the univariate analysis (Table-1). However, they could not be included in the multivariate analysis due to collinearity with other variables (herd size, pets on the farm, availability of feed storage space, and province). Herd size was classified into three categories: Small (<20 heads), medium (20-50 heads), and large (>50 heads). The risk of being seropositive was highest in cattle from large herds compared to small herds and medium herds (Table-1). This finding was similar to those of previous studies that reported a large herd size as a risk factor for BCoV infection [8,32]. This may be due to direct contact among a high density of animals within the herd. The odds ratio of being positive to BCoV in a herd containing >50 animals compared with a herd containing <50 animals was 1.39 (1.04-1.87, p=0.025) [32]. A greater probability of a visit by veterinarians and other people in large herds might be responsible for a higher risk of exposure to the pathogen, since BCoV can be harbored and indirectly transmitted by these people [33]. High stock density in large herds might be another reason related to a higher probability of being seropositive within these herds. In addition, difficulty dealing with the disease occurrence in large herds could be a further factor leading to the greater incidence of seropositivity.

In Japan, the spike gene that was found in canine respiratory coronavirus was similar to BCoV [34]. Kanno *et al*. [35] suggested that dogs can be a carrier of BCoV. Most of the participating dairy farmers had pets (dogs or cats) on their farms. Several CoVs have been detected in a wide variety of species and cross-species transmission is not uncommon [36]. However, there were no other farm animals in the participating herd. Thus, the present study focused only on the potential vectors for indirect transmission through pets. The transmission of BCoV is mainly through the fecal-oral route [4]. Available areas for storage of feed can protect the feed from contamination with the virus. The individual seroprevalence categorized by province is shown in Table-1. The variation in seroprevalence might be attributed to the different management practices adopted by farmers in each province [6,11]. Further studies may be required to clarify the four associations which could not be evaluated using multivariate analysis in the present study.

Individual animal history of diarrhea could not be evaluated in the present study due to a large number of missing values for this variable. Most farmers did not record it and could not remember illnesses in their animals. The history of diarrhea in the herd was not significantly associated with BCoV serological status based on Fisher’s exact test (Table-1). BCoV infection was more likely to be presented in a subclinical form, especially in re-infected animals. Stress, temperature, and host health status are important determinants of the infection [37,38]. Infected animals’ conditions can be worsened by co-infection with other common gastroenteric pathogens, such as *E. coli., Salmonella* spp., *C. parvum*, torovirus, and rotavirus [22,39]. In Norway, herds where there had been no introduction of new animals were more likely to be negative to BCoV compared to herds containing newcomers [40].

To prevent BCoV infection, vaccination [23,41], administration of antimicrobial agents [42], and improvement of animal environment and general health status can be introduced into a control program. Collaboration among all stakeholders should be established to prevent and control the disease, especially using knowledge provision and disease surveillance.

## Conclusion

BCoV seropositive dairy cattle were distributed throughout the western region of Thailand. The present study demonstrated that increased animal age was associated with a higher probability of being seropositive for BCoV. In contrast, using disinfectants to decontaminate the diarrhea stools in herds was associated with a lower risk of seroconversion compared with doing nothing and with cleaning with water. This study may improve the understanding of the factors associated with BCoV to design effective strategies for controlling BCoV in dairy herds.

## Authors’ Contributions

PA, PL, and NR: Supervised the study. PA: Responsible for study design and data collection. PL: Laboratory analysis. NR: Data analysis and the manuscript writing. SV: Conducted the study. All authors have read and approved the final manuscript.
